# Human gut-associated *Bifidobacterium* species salvage exogenous indole, a uremic toxin precursor, to synthesize indole-3-lactic acid via tryptophan

**DOI:** 10.1080/19490976.2024.2347728

**Published:** 2024-05-05

**Authors:** Cheng Chung Yong, Takuma Sakurai, Hiroki Kaneko, Ayako Horigome, Eri Mitsuyama, Aruto Nakajima, Toshihiko Katoh, Mikiyasu Sakanaka, Takaaki Abe, Jin-Zhong Xiao, Miyuki Tanaka, Toshitaka Odamaki, Takane Katayama

**Affiliations:** a Innovative Research Institute, Morinaga Milk Industry Co Ltd, Zama, Kanagawa, Japan; b Graduate School of Biostudies, Kyoto University, Kyoto, Japan; c Division of Nephrology, Endocrinology, and Vascular Medicine, Tohoku University Graduate School of Medicine, Sendai, Japan

**Keywords:** Indole, indole-3-lactic acid, tryptophan, *Bifidobacterium*, *trpB*, *aldh*

## Abstract

Indole in the gut is formed from dietary tryptophan by a bacterial tryptophan-indole lyase. Indole not only triggers biofilm formation and antibiotic resistance in gut microbes but also contributes to the progression of kidney dysfunction after absorption by the intestine and sulfation in the liver. As tryptophan is an essential amino acid for humans, these events seem inevitable. Despite this, we show in a proof-of-concept study that exogenous indole can be converted to an immunomodulatory tryptophan metabolite, indole-3-lactic acid (ILA), by a previously unknown microbial metabolic pathway that involves tryptophan synthase β subunit and aromatic lactate dehydrogenase. Selected bifidobacterial strains converted exogenous indole to ILA via tryptophan (Trp), which was demonstrated by incubating the bacterial cells in the presence of (2-^13^C)-labeled indole and l-serine. Disruption of the responsible genes variedly affected the efficiency of indole bioconversion to Trp and ILA, depending on the strains. Database searches against 11,943 bacterial genomes representing 960 human-associated species revealed that the co-occurrence of tryptophan synthase β subunit and aromatic lactate dehydrogenase is a specific feature of human gut-associated *Bifidobacterium* species, thus unveiling a new facet of bifidobacteria as probiotics. Indole, which has been assumed to be an end-product of tryptophan metabolism, may thus act as a precursor for the synthesis of a host-interacting metabolite with possible beneficial activities in the complex gut microbial ecosystem.

## Introduction

Gut microbial metabolism considerably affects host health. Among the diet-derived microbial metabolites, those from aromatic amino acids show variety in their structures and quantities, with both beneficial and harmful effects on host health being reported.^1,2^ Indole is one such metabolite that is formed from tryptophan (Trp) by the action of tryptophan indole-lyase (TIL), an enzyme widely distributed in gut microbes including species belonging to *Bacteroides*, *Clostridium*, and *Escherichia* genera.^3^ For microbes, indole acts as an intra- and interspecies signaling molecule that regulates growth, biofilm formation, antibiotic resistance, and virulence gene expression in the community.^3^ For the host, indole can exert both beneficial and harmful effects.^4^ It protected germ-free mice against chemically induced colitis by enhancing epithelial barrier function upon oral administration.^5^ Indole administration also alleviated non-steroidal and anti-inflammatory drug-induced enteropathy in a murine model.^6^ On the other hand, indole is a well-known uremic toxin precursor.^7,8^ After absorption by the intestine and sulfation in the liver, it circulates in the bloodstream. Indoxyl sulfate (IS) not only induces cell death in embryonic brain cells of mice^9^ but also causes podocyte injury, which contributes to the progression of kidney dysfunction, similarly to phenyl sulfate.^10^ This is a serious concern for patients with chronic kidney disease (CKD), as Trp is an essential amino acid for humans.

**Figure 1.**
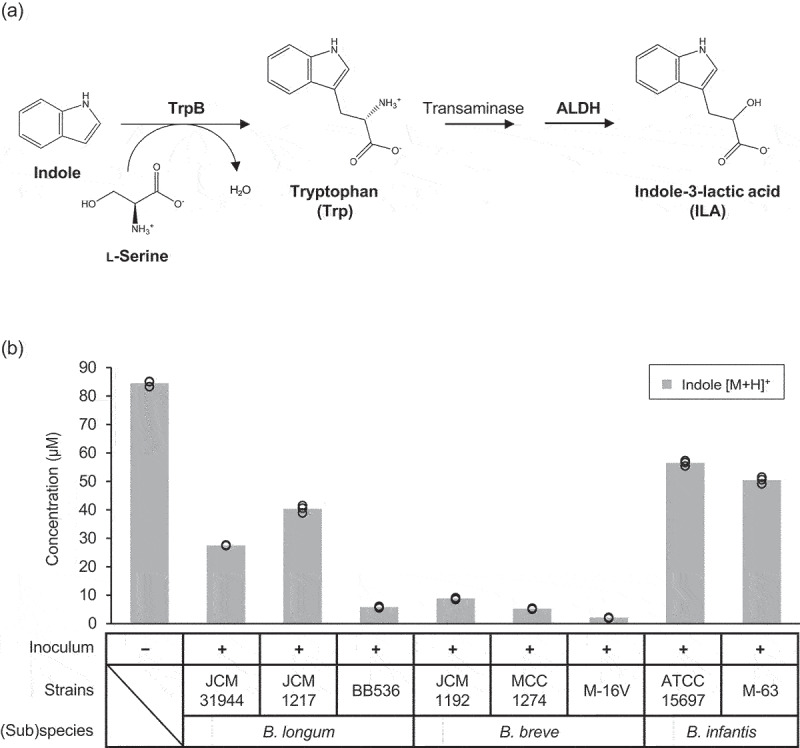
Consumption of exogenously added indole by selected bifidobacterial strains.

We have recently shown that human milk oligosaccharide-assimilating *Bifidobacterium* species possess an aromatic lactate dehydrogenase (ALDH) to produce a Trp metabolite, indole-3-lactic acid (ILA).^11–13^ ILA serves as a ligand for aryl hydrocarbon receptor and hydroxycarboxylic acid receptor 3, to help maintain intestinal and systemic immune homeostasis.^1^ We also demonstrated that *Bifidobacterium breve* upregulates its Trp metabolic genes including tryptophan synthase (*trpA* and *trpB*) and *aldh* when co-cultivated with human iPS-derived, monolayered intestinal epithelial cells.^14^ These studies suggest that certain *Bifidobacterium* species can shift their metabolic flow to *de novo* synthesis of ILA upon interaction with host cells. Given these results and assuming that TrpB catalyzes the condensation between l-serine and indole (discussed later), we hypothesized that bifidobacteria can incorporate exogenous indole into the Trp metabolic pathway to synthesize ILA (Figure 1a). Here, we uncover a previously unknown microbial metabolic pathway that salvages exogenous indole to synthesize ILA via Trp.

A. A possible route of TrpB- and ALDH-positive bifidobacteria to consume exogenous indole. B. The concentration of indole in the culture supernatants of selected *Bifidobacterium* strains grown in MRS medium supplemented with the spent medium of a TIL-positive *E. coli*. Non-inoculated medium was used as a control. Values of three biological replicates were plotted, while bars represent the mean. See Supplementary Table S5 and Source Data for plot data and statistical values.

## Materials and methods

### Chemicals

Indole, tryptophan, and indole-3-lactic acid (ILA) were purchased from Fujifilm Wako Pure Chemical Industries, Ltd (Tokyo, Japan). (2-^13^C)-Indole was obtained from Cambridge Isotope Laboratories, Inc (MA, USA). All other chemicals used were of analytical grade.

### Bacterial strains and culture conditions

The bacterial strains used in this study are listed in Supplementary Table S1 with their sources. Bifidobacteria were routinely grown in De Man, Rogosa, and Sharpe broth (MRS; BD Bioscience, Franklin Lakes, NJ, USA) supplemented with 0.05% (w/v) l-cysteine hydrochloride (Kanto Chemical, Tokyo, Japan) at 37°C under anaerobic conditions with Anaero Pack (Mitsubishi Gas Chemical, Tokyo, Japan). *Escherichia coli* K-12 MG1655, a wild-type strain with the TIL gene,^15^ was cultured in Luria-Bertani broth (LB; BD Biosciences, NJ, USA) at 28°C under aerobic conditions.

### Cultivation of Bifidobacterium strains in MRS broth supplemented with spent medium of E. coli

Overnight culture of *E. coli* K-12 MG1655 was centrifuged, and the supernatant was filtered through a 0.22-µm membrane (Millipore, MA, USA). The spent medium (*Ec*SM) was then mixed with an equal volume of l-cysteine-added MRS broth. The *Ec*SM-supplemented MRS broth was inoculated with an overnight culture of each *Bifidobacterium* strain with 3% (*v*/*v*), and the culture was incubated at 37°C under anaerobic conditions. Following 24 h incubation, the supernatant was obtained by centrifugation, to which an equal volume of methanol was added. The mixture was stored at −80°C until use.

### Fingerprinting of tryptophan metabolism using (2-^13^C)-indole

Bifidobacterial cells grown overnight in MRS broth were collected by centrifugation, washed twice with phosphate-buffered saline (PBS), and suspended again in PBS to give colony-forming units of ca. 2 × 10^9^ CFU/mL. To the suspension, l-serine and (2-^13^C)-indole were added to the final concentrations of 4 µM. After incubation at 37°C for 3 h under anaerobic conditions, the supernatants were collected and stored at −80°C until use.

### Quantification of tryptophan metabolites

A liquid chromatography-tandem mass spectrometry system (LC-MS/MS; Vanquish HPLC connected with TSQ-FORTIS, Thermo Fisher Scientific, MA, US) equipped with an XBridge® C8 column (4.6 × 150 mm, 5 μm; Waters Corporation, Milford, MA, USA) was used for the quantification of tryptophan metabolites. When the analysis included indole quantification, the culture supernatants were directly injected because of the volatility of indole, while in the other cases, they were vacuum-dried before reconstituting with 0.05% (*v*/*v*) formic acid containing 3-methyl-2-oxindole (MO) as an external standard at 0.1 µg/mL. Elution was carried out at a flow rate of 0.2 mL/min with a linear gradient between 0.05% (*v*/*v*) formic acid and methanol, in which methanol was increased from 2% to 90%. The target analytes were detected by electrospray ionization (ESI) in positive ion mode with multiple reaction monitoring (MRM) methods (Supplementary Table S2). Standard curves were created using known concentrations of respective compounds. The amount of MO was used for standardization.

### Construction of insertional mutants

The gene for tryptophan synthase β subunit (*trpB*) of *B. longum* JCM 31944^16^ and the genes for aromatic lactate dehydrogenases (*aldh*) of *B. breve* MCC1274 and *M*-16 V were disrupted by suicide plasmid-mediated single crossover recombination events. *E. coli* DH5α was used as a gene manipulation host, while an In-Fusion cloning HD kit (Clontech Laboratories, Inc., CA, USA) was used for plasmid construction. The mutant construction scheme was the same as described previously.^17^ In brief, for *trpB* disruption, the PCR-amplified internal region of the *trpB* gene was ligated with the *SacI-* and *NcoI*-digested pKKT427^18^ fragment containing the pUC *ori* and the spectinomycin-resistance gene. The resulting suicide plasmid was introduced into *B. longum* JCM 31944 by electroporation, and the disruptant was screened on Gifu anaerobic medium (GAM) agar plates (Nissui Pharmaceutical Co., Ltd., Tokyo, Japan) containing 30 µg/mL spectinomycin. Disruption of *aldh* was similarly performed except that pMSK209^17^ was used as a suicide vector. The PCR-amplified internal region of *aldh* of *B. breve* MCC1274 and M-16 V strains were separately ligated with the *NsiI*-digested pMSK209 fragment containing the pUC *ori* and the chloramphenicol-resistance gene. The resulting plasmids were introduced into the corresponding strains by electroporation, and the mutants were selected on GAM agar plates containing 10 µg/mL chloramphenicol. Integration of the plasmid into the targeted locus of the genome (*trpB* or *aldh*) was confirmed by genomic PCR, for which a primer pair designed to anneal outside the region used for homologous recombination was used. All primers used for mutant construction and confirmation are listed in Supplementary Table S3. The *aldh* mutant of *B. longum* JCM 31944 was constructed in our previous study.^13^ The generated mutants were used for (2-^13^C)-indole-based fingerprinting as mentioned above.

### Gene distribution analysis

The sequence-completed genomes of bacteria that are specified as those associated with *Homo sapiens* as the host in the NCBI database (https://www.ncbi.nlm.nih.gov/genome/browse/) were selected for the analysis. From the data available in October 2023, multi-fasta files of amino acid sequences of 960 species/subspecies represented by 11,943 genomes were downloaded and utilized as a local database for the subsequent homolog analysis. The taxonomic names and the number of genomes examined for each taxon are listed in Supplementary Table S4.

The presence of the homologs of TrpB (UniProt ID: P0A879), FldH (CLOSPO_00316aldh),^19^ and ALDH (BL105A_0985)^12^ was examined using MMseqs2 (v.14.7e284).^20^ The protein sequences encoded in each of the genomes were transformed into a database using the ‘createdb’ function, and ‘easy-search’ was employed for the protein BLAST analysis using a parameter for the highest sensitivity (7.5). The search results were subsequently processed using the Pandas library in Python 3.11. Hits with both similarity and coverage of ≥50% were tallied as positive.

### Statistical analysis

Student’s *t*-test and Dunnett’s test were used for two- and multiple-group comparisons, respectively, and *p* values less than 0.05 were considered statistically significant. *p* values are reported in Supplementary Tables S5–S8.

## Results and discussion

As a proof-of-concept study, we first examined whether bifidobacteria can consume exogenously added indole. We used strains belonging to *Bifidobacterium longum* subsp. *longum* (*B. longum*), *Bifidobacterium longum* subsp. *infantis* (*B. infantis*), and *B. breve*, as these species specifically harbor the *aldh* gene among gut microbes^12^ and are reported to colonize the human gut with relatively high abundance across different stages of life.^21^ All strains are isolated from human feces. Selected *Bifidobacterium* strains were grown in MRS medium supplemented with the spent medium of a TIL-positive *E. coli* strain. The obtained supernatants were then quantified for indole. The results showed that all tested strains consumed exogenous indole to different extents, ranging from 34% to 98% reduction (Figure 1b, Supplementary Table S5).

**Figure 2.**
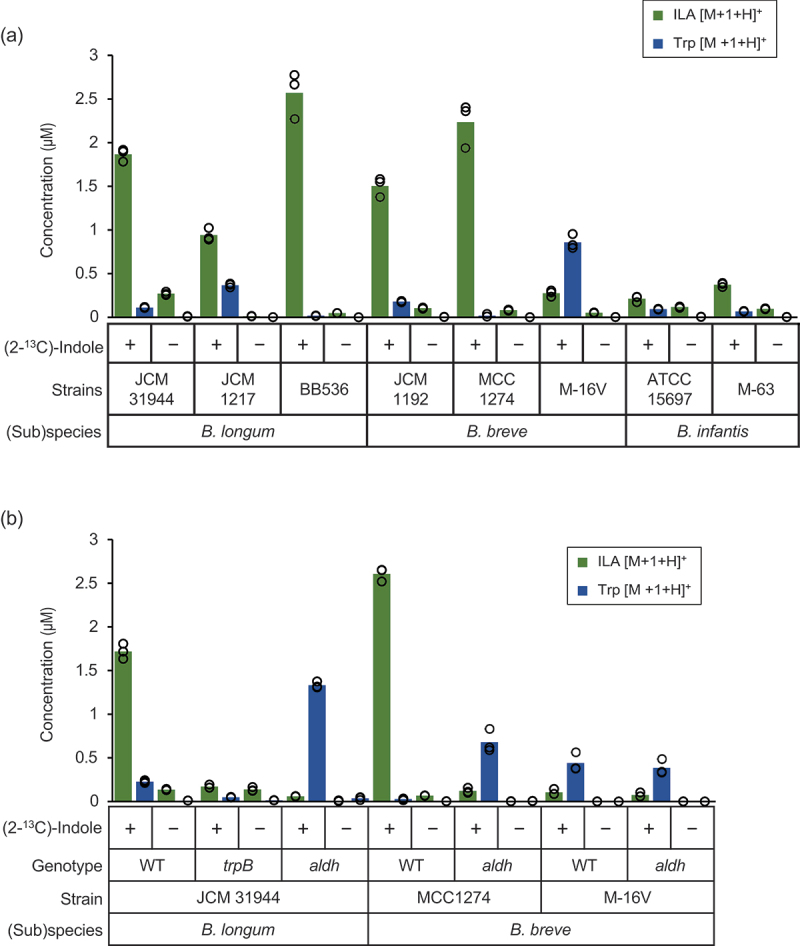
Bifidobacterial bioconversion of indole to ILA occurs with Trp as an intermediate.

We then incubated bifidobacterial cell suspensions in the presence and absence of 4 µM of substrates, (2-^13^C)-indole and l-serine, and quantified Trp and ILA in the supernatant. Although we measured both *m*/*z* of [M + H]^+^ and [M + 1 + H]^+^ ion peaks of the target compounds, we refer to the data obtained for *m*/*z* of [M + 1 + H]^+^ molecules only, as we did not find any remarkable difference in the concentrations of [M + H]^+^ compounds between the substrate-added and non-added samples (Supplementary Table S6). Upon incubation with the substrates, the total amount of Trp plus ILA significantly increased by 2.5- to 100-fold as compared to those incubated without the substrates, indicating that the cells utilized exogenous indole to synthesize Trp and ILA (Figure 2a and Supplementary Table S6). All tested strains produced ILA as the main product, except for *B. breve M*-16 V, which produced a larger amount of Trp than ILA (0.86 μM vs. 0.28 μM). The highest level of ILA production was observed for *B. longum* BB536 at 2.57 μM. Overall, strains belonging to *B. longum* and *B. breve* showed higher indole bioconversion ability than those belonging to *B. infantis*. Notably, these results also suggest the presence of specific transporters for the excretion of ILA and Trp in these bacteria.

a and b, Concentrations of ILA (green bars) and Trp (blue bars) with *m*/*z* of [M + 1 + H]^+^ values in the supernatants of cell suspensions after incubation for 3 h with and without 4 μM of (2-^13^C)-indole plus l-serine. Values of three biological replicates were plotted, with bars representing the mean. See Supplementary Tables S6 (for a), S7 and S8 (for b), and Source Data (for a and b) for plot data and statistical values. a, Overnight cultures of the selected bifidobacterial strains grown in MRS medium were centrifuged, and the harvested cells suspended in PBS were used for the reactions. b, Cell suspensions of WT, t*rpB*, and *aldh* strains were similarly prepared and used for the reactions.

We then inactivated *trpB* or *aldh* by a single crossover recombination^17^ to identify the responsible pathway for indole bioconversion (Figure 1a). Cell suspensions of wild-type (WT) and mutant strains were similarly incubated with and without (2-^13^C)-indole and l-serine. No remarkable change was detected in the concentrations of *m*/*z* of [M + H]^+^ compounds between the substrate-added and non-added samples, and thus, we again refer to the compounds with *m*/*z* of [M + 1 + H]^+^ only (Supplementary Tables S7 and S8). In the supernatant of *B. longum* JCM 31,944 *trpB* mutant incubated with the substrates, small amounts of Trp and ILA were detected (Figure 2b). The total amount of the metabolites obtained for the mutant was not only ninefold less than that detected for the WT incubated with the substrates (0.22 vs. 1.95 μM) but also was comparable with the amount obtained for the mutant incubated without the substrates (0.22 vs. 0.15 μM) (Supplementary Tables S7 and S8), indicating that the *trpB* mutant was incapable of incorporating indole into the bioconversion flow. By contrast, the *aldh* mutant of the same strain incorporated indole into the metabolic route, as the total amount of ILA plus Trp reached 1.39 μM (72% of WT). However, the metabolic flow stopped at Trp in the mutant. The amount of Trp corresponded to 77% of ILA produced by the WT incubated under the same conditions (1.33 vs. 1.72 μM). Similarly, disruption of *aldh* had a pronounced effect on ILA production by *B. breve* MCC1274. The *aldh* mutant produced a 22-fold lower amount of ILA (0.12 μM vs. 2.61 μM) and a 25-fold higher concentration of Trp (0.68 vs. 0.03 μM) than the WT strain when incubated in the presence of the substrates. By contrast, for *B.* *breve* M-16 V, no difference in the metabolite profiles was observed between the WT and *aldh*-mutant cells incubated with the substrates. The small amount of ILA detected in the supernatant of *B.* *breve* M-16 V might have been synthesized by a canonical lactate dehydrogenase (LDH), as seen in *Lactobacillus* species.^22^ Although the total amount of ILA plus Trp differed by twofold (1.1 μM in Figure 2a vs. 0.5 μM in Figure 2b), a similar indole bioconversion profile was obtained in the two separate experiments for *B.* *breve* M-16 V WT. Taken together, our study revealed that certain *Bifidobacterium* strains can incorporate exogenous indole to synthesize Trp and/or ILA with varied efficiency, in which TrpB and Aldh or canonical LDH could be involved. It is not surprising that the reaction occurs, as indole rapidly diffuses through the bacterial cell membrane due to its nonpolar nature at neutral pH.^23^

Finally, using the NCBI database, we examined the prevalence of TrpB and ALDH homologs in 11,943 genomes representing 960 bacterial (sub)species associated with humans (Supplementary Table S4). FldH of *Clostridium sporogenes*,^19^ the isozyme of ALDH, was also included as a query sequence in the analysis. TrpB, FldH, and ALDH homologs were found in the genomes of 9824, 10, and 95 strains, respectively. Neither TrpB/FldH-double positive nor FldH/ALDH-double positive strains were found. *C. sporogenes*, which was not included in the retrieved database, was found to be TrpB negative by a manual BLAST analysis. By contrast, all 95 of the ALDH-positive strains possessed TrpB homologs and belonged to the *Bifidobacterium* genus comprising 16 (sub)species and 3 unclassified species (Supplementary Table S9). Remarkably, 92 out of the 95 double-positive strains were human gut-associated bifidobacterial (sub)species (*B. breve*, *B. longum*, and *B. infantis*, *Bifidobacterium bifidum*, *Bifidobacterium angulatum*). Among the 10 *Lactobacillus* species, some of which may produce ILA using a canonical LDH,^22^ only *Lactobacillus helveticus* possessed TrpB homolog.

Tryptophan synthase is generally comprised of two polypeptide chains TrpA and TrpB to form a heteromeric complex.^24^ TrpA releases indole from indole-3-glycerol phosphate that is formed by the action of TrpC (indole-3-glycerol-phosphate synthase), and the resulting indole is sent to the catalytic center of TrpB through a tunnel-like structure in the TrpAB complex. Indole is thus sequestered from other components before being condensed with l-serine. Based on this reaction mechanism, tryptophan synthase had not been considered to accept exogenous indole for its reaction.^25^ However, the indole – Trp conversion reaction has been reported for one archaeon *Thermococcus kodakarensis* that has two distinct TrpB chains (TrpB1 and TrpB2), one of which does not form a complex with TrpA.^25^ As TrpB of bifidobacteria constitutes a chimeric polypeptide with TrpC, its tertiary structure (subunit assembly) might be different from that of the typical tryptophan synthase most bacteria possess.

Our study was conducted under controlled *in vitro* conditions and did not consider the environmental factors encountered by bifidobacteria in the complex microbial ecosystem in the gut. In addition, there may be different indole bioconversion pathways that have not been explored in this study. Nonetheless, it is interesting to note that a previous study reported a decreased serum IS concentration for dialysis patients with oral administration of *B. longum*.^26^ Our study revealed a previously unknown bifidobacteria-specific metabolic pathway that salvages exogenous indole to synthesize an immunomodulatory Trp metabolite, ILA. The results provide the groundwork aimed at a probiotic intervention with a novel therapeutic viewpoint, especially for CKD patients. It is imperative to foster a comprehensive understanding of how this metabolic pathway potentially works in human physiology. Future clinical studies should be designed to consider the indole bioconversion pathway, and their outcomes should be interpreted accordingly, as indole may serve as both an end-product and a precursor in tryptophan metabolism within the gut ecosystem.

## Supplementary Material

Supplemental Material
